# Hypercapnic Ventilatory Response in the Weaning of Patients with Prolonged Mechanical Ventilation

**DOI:** 10.1155/2017/7381424

**Published:** 2017-10-30

**Authors:** Chung-Shu Lee, Ning-Hung Chen, Li-Pang Chuang, Chih-Hao Chang, Li-Fu Li, Shih-Wei Lin, Hsiung-Ying Huang

**Affiliations:** ^1^Department of Thoracic Medicine, Chang Gung Memorial Hospital, Linkou, No. 5, Fu-Hsing St., Kueishan Dist., Taoyuan City, Taiwan; ^2^Department of Respiratory Therapy, Chang Gung University, Taoyuan City, Taiwan; ^3^Department of Pulmonary and Critical Care Medicine, Xiamen Chang Gung Hospital, Xiamen City, China

## Abstract

**Objective:**

To investigate whether hypercapnic ventilatory response (defined as the ratio of the change in minute ventilation [$\Delta {\dot{V}}_{E}$] to the change in end-tidal partial pressure of carbon dioxide [Δ*P*_ETCO_2__]) is a predictor of successful weaning in patients with prolonged mechanical ventilation (PMV) and to determine a reference value for clinical use.

**Methods:**

A hypercapnic challenge test was performed on 32 PMV subjects (average age: 74.3 years ± 14.9 years). The subjects were divided into two groups (i.e., weaning successes and weaning failures) and their hypercapnic ventilatory responses were compared.

**Results:**

PMV subjects had an overall weaning rate of 68.8%. The weaning-success and weaning-failure groups had hypercapnic ventilatory responses ($\Delta {\dot{V}}_{E}/\Delta P_{{ETCO}_{2}}$) of 0.40 ± 0.16 and 0.28 ± 0.12 L/min/mmHg, respectively (*P* = .036). The area under the receiver operating characteristic curve was 0.716 of the hypercapnic ventilatory response, and the practical hypercapnic ventilatory response cut-off point for successful weaning was 0.265 with 86.4% sensitivity and 50% specificity.

**Conclusions:**

PMV subjects who failed weaning had a lower hypercapnic ventilatory response than successfully weaned subjects. However, the prediction capacity of this test, assessed by the area under the receiver operating characteristic (ROC) curve, poorly predicted weaning outcome.

## 1. Introduction

The use of a mechanical ventilator for more than 21 days is defined as prolonged mechanical ventilation (PMV) [1], and a longer duration of mechanical ventilation is associated with higher medical costs and an increased risk of ventilator-associated pneumonia [2]. Patients with PMV who fail weaning generally have longer hospital stays and worse outcomes [3] compared with patients who are successfully weaned.

In Taiwan, a special facility called a respiratory care center (RCC), a step-down care unit distinct from an intensive care unit designed to aid with weaning, is used for patients with PMV with a stable hemodynamic status who need specialized respiratory care. Among patients with PMV in the RCC, reduced respiratory central drive is an important pathophysiological factor responsible for failed weaning [4].

Respiratory drive can be evaluated by airway occlusion pressure or hypercapnic ventilatory response [5]. Airway occlusion pressure (*P*_0.1_), the negative airway pressure at 0.1 seconds after inspiration is occluded, is measured using a mechanical ventilator and requires a medical doctor and respiratory therapist to evaluate respiratory center motor output. Occlusion pressure [6, 7] and its ratio to maximal inspiratory pressure [8] are useful predictors of successful weaning. In addition to the *P*_0.1_ value, the change in *P*_0.1_ according to hypercapnic status has also been suggested as an index of respiratory drive [5]. In patients with PMV with brain stem lesions, successful weaning has been associated with higher changes in *P*_0.1_ to hypercapnic stimulation [9]. Another tool used to evaluate respiratory drive is hypercapnic ventilatory response (the ratio of $\Delta {\dot{V}}_{E}$ to Δ*P*_ETCO_2__), which is assessed by performing the hypercapnic challenge test using the CO_2_ rebreathing method developed by Read [10, 11]. The hypercapnic drive concept is initiated from brain tissue CO_2_, which has usually been replaced by arterial or end-tidal CO_2_, as an index of the CO_2_ stimulus over the medullary chemoreceptors [12], and the ventilatory response replies to the CO_2_ stimulus [13]. Hypercapnic ventilatory response has been studied in spontaneous breathing trials and extubation [14], duration of weaning [15], central sleep apnea [16], and relatives of patients with obesity hypoventilation syndrome [17].

To date, no previous clinical study has evaluated respiratory drive measured by a hypercapnic ventilatory response in patients with PMV. Therefore, the aim of this study was to determine whether a hypercapnic ventilatory response may be a predictor of successful weaning in patients with PMV and to determine a reference value for clinical use.

## 2. Materials and Methods

### 2.1. Patients

This study was approved by the Institutional Review Board (number 101-3531A3) of our hospital.

Subjects were enrolled from a 24-bed RCC located in a 3800-bed tertiary medical center containing 350 ICU beds. Patient resources included medical, neurological, surgical, neurosurgery, burn, and trauma ICUs and a coronary care unit. Few patients came from other hospitals.

**Figure 1:**
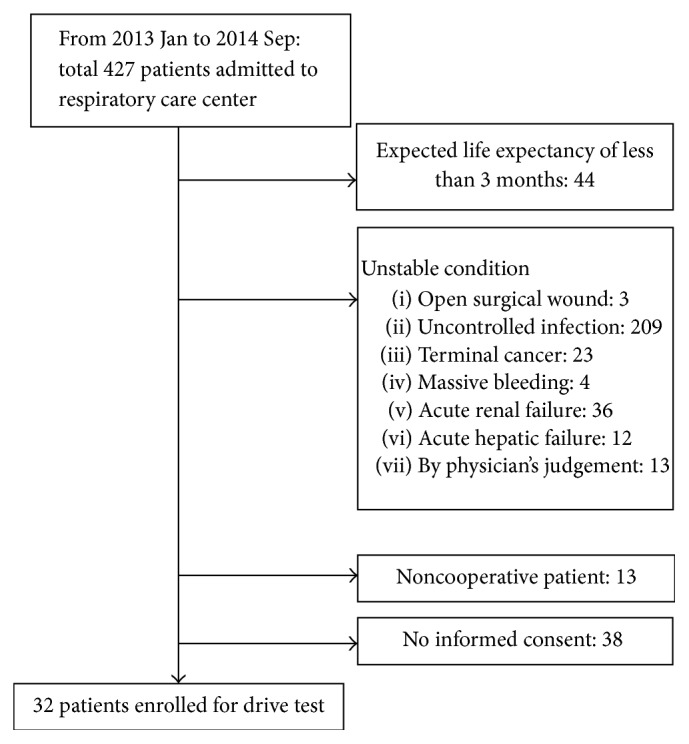
Diagram of enrolled patients with hypercapnic ventilatory response in respiratory care center.

The RCC is a step-down, subacute care facility after ICU. The purpose of the RCC is to care for patients who are on mechanical ventilation for more than 21 days and for patients who experience difficulty in weaning from mechanical ventilation. All patients who were transferred to the RCC from January 2013 to September 2014 were screened for inclusion in this study. Eligibility criteria included >18 years of age, respiratory failure with the use of mechanical ventilation longer than 21 days, hemodynamic stability without the use of inotropic agents or sedatives, and the use of spontaneous ventilator mode with an *F*_IO_2__ < 40%. We had ruled out pulmonary edema, myocardial dysfunction, ventilator-associated pneumonia, and delirium status before RCC admission. The exclusion criteria were expected life expectancy of less than 3 months; an unstable clinical condition including terminal cancer, massive bleeding, acute renal failure, acute hepatic failure, or any other condition the physician judged to be unstable; and refusal to provide informed consent. A diagram of recruitment is shown in Figure 1.

### 2.2. Weaning Process

The weaning process was initiated when the patients were judged to be ready for weaning. If the patients had an unstable hemodynamic condition, were using inotropic agents, had unstable vital signs (body temperature > 38°C; heart rate > 140 beats per minute; respiratory rate > 30 breaths per minute), or did not have a low ventilator setting (*F*_IO_2__ > 40%; PEEP > 8 cmH_2_O), they were considered to be unsuitable for weaning. Either a step-by-step reduction in pressure support mode of the mechanical ventilator or an increased duration of spontaneous breathing was the main indicator for the weaning process with daily evaluations, after which the weaning protocol [9] was initiated and supervised by a respiratory therapist. The cornerstone of the protocol was to shift from full ventilatory support to 24-hour unassisted and spontaneous breathing. Shifting from full to partial ventilator support was achieved by reducing the level of pressure support ventilation or synchronized intermittent mandatory ventilation. Among tracheostomized patients, spontaneous breathing trials were performed by using a Venturi tracheostomy O_2_ mask, with gradual increases in the duration of the spontaneous breathing trials. We had measured the maximal inspiratory pressure by pressure gauge. After the patient was in stable status with smooth respiratory pattern, we occluded the inspiratory tube for 20 seconds manually. During the occlusion, the gauge would detect several inspiratory pressures, and the highest level of inspiratory pressure was the maximal inspiratory pressure. Weaning success was defined as liberation from mechanical ventilation for 5 continuous days. Weaning failure was defined as the need for mechanical ventilation even after the weaning process and clinical adjustment.

### 2.3. Hypercapnic Challenge Test for Ventilatory Response

The modified Read rebreathing method [10] was used as the hypercapnic challenge test. All patients in the trial were placed in the supine position with a pulse belt, continuous pulse oximeter, and electrocardiographic monitoring during the hypercapnic challenge test. A respiratory therapist and a physician were at the bedside during the entire test. Respiratory rate, blood pressure, and heart rate were measured 20 minutes prior to the start of the test. A bedside capnograph (Capnostream® 20, Oridion, USA) and a pneumotachograph (PowerLab® Systems 16/30, AD Instruments, New Zealand) recorded data with Lab Chart Pro software and were directly connected to the patient's endotracheal or tracheostomy tube.

*P*_ETCO_2__, flow, and respiratory rate were recorded simultaneously using a PowerLab system. The air inlet of the ventilator was connected to the central air source, while a cylinder containing 10% CO_2_ and 90% O_2_ gas was connected to the O_2_ inlet. *P*_0.1_ was measured by pressing the *P*_0.1_ button on the ventilator (Drager Evita II Dura, Drager Medical AG & Co. Kga A, Lubeck, Germany). Real-time minute ventilation was calculated using the PowerLab system. The patient breathed spontaneously while the trigger sensitivity was set to minimum (2 L/sec) under a pressure support mode of mechanical ventilation with a positive end expiratory pressure up to 8 cm H_2_O. Following the observation time, hypercapnic challenge was increased by adjusting the inlet flow toward the 10% CO_2_ mixture using the *F*_IO_2__ button on the ventilator in 5 mmHg increments from the baseline *P*_ETCO_2__ level. The scheduled observation time was terminated when the *P*_ETCO_2__ reached 70 mmHg or the test time reached 3 minutes to avoid prolonged respiratory acidosis. *P*_0.1_, respiratory rate, minute ventilation volume, blood pressure, and *P*_ETCO_2__ were collected for each 5 mmHg *P*_ETCO_2__ increment from the *P*_ETCO_2__ baseline level. Measurements ceased immediately if any of the following conditions occurred: saturation O_2_ < 90%, heart rate > 140 beats or <55 beats per minute, systolic blood pressure > 180 or <90 mmHg, a change in consciousness, or if the patient felt anxious or became agitated.

### 2.4. Statistical Analysis

Continuous variables were expressed as mean ± SD, and categorical data were expressed as frequency and percentage. Clinical characteristics and baseline respiratory assessments were compared using the independent two sample *t*-test or chi-square test. A cut-off value was determined by receiver operating characteristic curve (ROC) analysis. Data were analyzed using SPSS software version 18.0 (SPSS Inc., Chicago, IL) and a *P* value < .05 was considered statistically significant.

## 3. Results

A total of 427 patients were screened, of whom 32 were enrolled in the trial. Their characteristics, demographics, clinical, and physiological variables are shown in Table 1.

All 32 subjects were classified into either weaning-success or weaning-failure groups. The overall weaning rate was 68.8%. The subjects in the weaning-failure group were significantly older than those in the weaning-success group (82.5 years ± 10.3 years versus 70.6 years ± 15.3 years, resp., *P* = .034). The duration of ventilation-use days till drive test was also significantly longer in the weaning-failure group compared with the weaning-success group (35.5 days ± 6.6 days versus 29.5 days ± 5.3 days, resp., *P* = .011). However, there were no significant differences in gender, body mass indices, percentage of patients from the medical ICU, number of tracheostomies, or APACHE II scores between the two groups. There were total ten weaning-failure patients, and six of them died; three had been transferred to respiratory care wards with long-term mechanical ventilator dependent status. Only one was judged as weaning failure in RCC but was successfully weaned when the patient was transferred to the ordinary ward. There was also no significant difference in respiratory function between the two groups except hypercapnic ventilatory response (Table 2).

**Figure 2:**
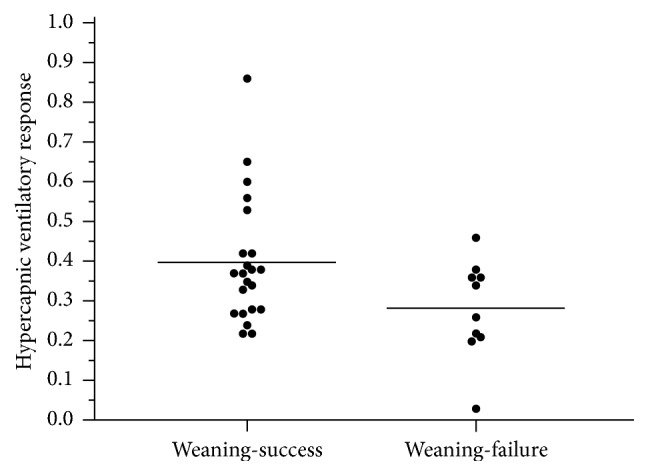
Plotted graph with hypercapnic ventilatory response of weaning-success and weaning-failure groups; *P* = .036.

The hypercapnic ventilatory response ($\Delta {\dot{V}}_{E}$/Δ*P*_ETCO_2__) was 0.40 L/min/mmHg ± 0.16 L/min/mmHg in the weaning-success group compared with 0.28 L/min/mmHg ± 0.12 L/min/mmHg in the weaning-failure group (*P* = .036) (Figure 2). The hypercapnic drive response (Δ*P*_0.1_/Δ*P*_ETCO_2__) was 0.33 L/min/mmHg ± 0.22 L/min/mmHg in the weaning-success group compared with 0.34 L/min/mmHg ± 0.21 L/min/mmHg in the weaning-failure group (*P* = .991).

**Figure 3:**
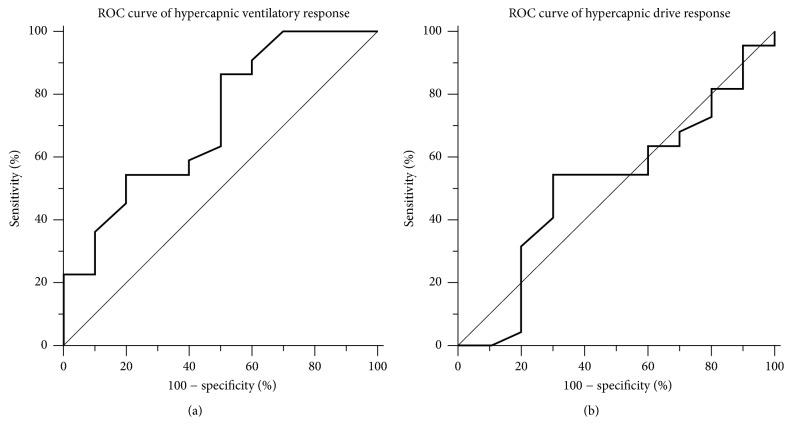
(a) Receiver operating characteristic (ROC) curve of hypercapnic ventilatory response and area under the curve (AUC): 0.716; (b) receiver operating characteristic (ROC) curve of hypercapnic drive response and area under the curve (AUC): 0.486.

The areas under the ROC curve (Figure 3) were 0.716 and 0.486 for the hypercapnic ventilatory response and hypercapnic drive response, respectively. The optimal hypercapnic ventilatory response cut-off point to predict weaning success was 0.265 with a sensitivity of 86.4% and a specificity of 50%.

After controlling with confounding factors, multivariate analysis of possible factors with weaning success was performed (Table 3), and the ventilation-use days till drive test is with significance as predictor of weaning success.

## 4. Discussion

This study investigated whether hypercapnic ventilatory response (defined as the ratio of the change in minute ventilation [$\Delta {\dot{V}}_{E}$] to the change in end-tidal partial pressure of carbon dioxide [Δ*P*_ETCO_2__]) was a predictor of successful weaning in patients with prolonged mechanical ventilation (PMV). Our results revealed a significant difference in hypercapnic ventilatory response between subjects with PMV who were successfully weaned compared with those who failed weaning.

Hypercapnic ventilatory response has been used to predict weaning outcomes in ICU patients on short durations of mechanical ventilation [14, 18] and in various other populations [16–18]. However, to the best of our knowledge, this study was the first to focus on subjects with PMV. Our results were similar to those published by Raurich et al. [15], in which a higher hypercapnic ventilator response was more likely to predict successful weaning. However, their study focused on weaning duration (time from the first spontaneous breathing trial to the day of successful weaning); that is, subjects with a duration longer than 7 days were compared with those with a duration shorter than 7 days. By contrast, we compared subjects with and without successful weaning, and all of our enrolled subjects had used a ventilator for more than 21 days.

Chemosensitivity determined with the hypercapnic ventilatory response provides a guide of the integrity of the respiratory system. Any impairment of the respiratory system (ventilatory or neuromuscular apparatus and metabolic control) can reduce the hypercapnic ventilatory response [19]. ICU patients ready for weaning from mechanical ventilation but failing a spontaneous breathing trial have lower carbon dioxide (CO_2_) response than successfully weaned patients [6, 14, 18, 20]. Patient's age [21] and muscular weakness induced by mechanical ventilation [22] may contribute to the low hypercapnic ventilatory response. Chemoreceptor blunting could also be a contributing factor responsible for weaning failure in some patients [23]. Other reasons might explain the low hypercapnic ventilatory response including patients who failed weaning developed worsening pulmonary mechanics [23], with a lung volume close to the total lung capacity and the diaphragm unable to act as an inspiratory muscle [24].

One of the most common concerns when measuring respiratory drive is the effect of the respiratory muscles on respiratory drive. In general, once the respiratory muscles are affected by trauma, dysfunction of the neural system, oxidative stress, or cytokines (secondary to inflammatory or immune reactions), the affected respiratory muscles may not generate enough drive. In order to avoid such a confounding factor, we shortened the hypercapnic challenge test time to 3 minutes in our study.

Hypoventilation is an additional issue among PMV patients that is still under debate. In our study, the baseline *P*_aCO_2__ level in the weaning-failure group was higher than that in the weaning-success group (44.9 ± 8.0 versus 38.9 ± 8.1 mmHg, *P* = .059). It is worthwhile to note that poor drive related to alveolar hypoventilation may occur in the weaning-failure group and result in a failed liberation from the ventilator; that is, poor respiratory drive can lead not only to hypoventilation but also to poor outcome.

We chose *P*_ETCO_2__ instead of *P*_aCO_2__ because the value of *P*_ETCO_2__ correlated with that of *P*_aCO_2__, and there was a gap difference between *P*_ETCO_2__ and *P*_aCO_2__. In our study, we measured baseline *P*_ETCO_2__ and *P*_aCO_2__ with 37.1 ± 6.9 and 40.8 ± 8.4 mmHg, respectively. Linear regression analysis showed a good correlation (*r* = 0.725) between *P*_ETCO_2__ and *P*_aCO_2__. In addition, Jones et al. [25] have validated the correlation between *P*_ETCO_2__ and *P*_aCO_2__ with tidal volume, and several studies have also shown that *P*_ETCO_2__ could represent *P*_aCO_2__ under certain conditions [26, 27]. The hypercapnic ventilatory response was calculated using the change in minute ventilation divided by the change in *P*_ETCO_2__, and although we did not obtain the absolute value of *P*_aCO_2__, we felt that it was feasible to use the change in *P*_ETCO_2__ instead of the change in *P*_aCO_2__ as their values appeared to correlate. In addition, hypercapnic challenge using *P*_ETCO_2__ has been applied to patients with brain stem lesions with good success [9].

The duration of ventilator use was higher in our weaning-failure group compared with our weaning-success group (35.5 days ± 6.6 days versus 29.5 days ± 5.3 days, *P* = .011). This finding is similar to that reported by Hermans et al. in which an increased duration of mechanical ventilation was associated with a decline in diaphragmatic force [28]. Respiratory muscle weakness is an important risk factor for delayed weaning [4], and PMV causes changes in both diaphragmatic structure and function [29].

The subjects in our weaning-failure group were older than those in the weaning-success group (82.5 years ± 10.3 years versus 70.6 years ± 15.3 years, *P* = .034). Some studies [30, 31] have reported that advanced age is a risk factor for weaning failure. Comorbidities are common in the elderly and a lower comorbidity burden is more likely to result in successful weaning from mechanical ventilation [30]. As for “ventilation-use days till hypercapnic challenge test” being associated with weaning success, once the patient was in unstable condition, we would not proceed to assess the weaning. Weaning failure may be associated with previously more medical conditions which resulted in more ventilation-use days till hypercapnic challenge test. Although the hypercapnic ventilatory response is not the significant predictor after logistic regression analysis, we could see its trend to be an adjuvant predictor.

Our study had several limitations including its small sample size. Future studies involving larger numbers of subjects are needed to confirm our results.

In conclusion, the results of our study showed that PMV patients who failed weaning had a lower hypercapnic ventilatory response than successfully weaned patients. However, the prediction capacity of this test, assessed by the area under the ROC curve, poorly predicted weaning outcome.

## Figures and Tables

**Table 1:** Clinical characteristics of enrolled subjects.

Characteristic	All (*n* = 32)	Weaning success (*n* = 22)	Weaning failure (*n* = 10)	*P* value
Age, mean ± SD years	74.3 ± 14.9	70.6 ± 15.3	82.5 ± 10.3	.034^*∗*^
Male, number (%)	19 (59.4%)	14 (63.6%)	5 (50%)	.467
Height, mean ± SD cm	160.1 ± 10.7	161.6 ± 11.1	156.6 ± 9.3	.222
Body mass index, mean ± SD kg/m^2^	22.7 ± 4.5	22.8 ± 4.8	22.5 ± 3.8	.848
Transfer from medical ICU, number (%)	28 (87.5%)	20 (90.9%)	8 (80.0%)	.387
Tracheostomy, number (%)	8 (25.0%)	5 (22.7%)	3 (30.0%)	.660
APACHE II score	20.7 ± 6.1	20.3 ± 6.5	21.5 ± 5.2	.617
Ventilation-use days till drive test, mean ± SD day	31.4 ± 6.3	29.5 ± 5.3	35.5 ± 6.6	.011^*∗*^
Hemoglobin, mean ± SD g/dL	9.86 ± 1.41	9.82 ± 1.56	9.95 ± 1.08	.811
Albumin, mean ± SD g/dL	2.81 ± 0.52	2.84 ± 0.52	2.76 ± 0.54	.710
TSH, mean ± SD *μ*IU/mL	2.49 ± 1.89	2.19 ± 1.99	3.11 ± 1.59	.215
Free-T4, mean ± SD ng/dL	1.14 ± 0.32	1.12 ± 0.28	1.19 ± 0.41	.551
Reason for admission				
Pneumonia, number (%)	15 (53.1%)	8 (36.4%)	7 (70.0%)	.077
AECOPD, number (%)	2 (6.3%)	2 (9.1%)	0 (0.0%)	.325
Congestive heart failure, number (%)	4 (12.5%)	2 (9.1%)	2 (20.0%)	.387
Myocardial infarction, number (%)	1 (3.1%)	1 (4.5%)	0 (0.0%)	.493
Cerebrovascular disease, number (%)	6 (18.8%)	6 (27.3%)	0 (0.0%)	.067
Ventricular tachycardia, number (%)	1 (3.1%)	1 (4.5%)	0 (0.0%)	.493
Urinary tract infection, number (%)	1 (3.1%)	1 (4.5%)	0 (0.0%)	.493
Burn, number (%)	1 (3.1%)	0 (0.0%)	1 (10.0%)	.132
Gastrointestinal bleeding, number (%)	1 (3.1%)	1 (4.5%)	0 (0.0%)	.493

APACHE II: Acute Physiology and Chronic Health Evaluation II; ventilation-use days till drive test: the duration time from the first day of mechanical ventilation use to the day of hypercapnic challenge test; AECOPD: chronic obstructive pulmonary disease with acute exacerbation; *∗* denotes statistical significance with *P* < .05.

**Table 2:** Respiratory function assessment.

	All (*n* = 32)	Weaning success (*n* = 22)	Weaning failure (*n* = 10)	*P* value
Baseline arterial blood gases				
pH	7.47 ± 0.06	7.47 ± 0.07	7.47 ± 006	.917
*P*_aCO_2__, mmHg	40.8 ± 8.4	38.9 ± 8.1	44.9 ± 8.0	.059
*P*_aO_2__, mmHg	108.7 ± 30.1	112.2 ± 31.9	101.0 ± 25.6	.336
HCO_3_^−^, mmol/L	29.1 ± 6.4	27.8 ± 5.1	31.9 ± 8.3	.091
*P*_aO_2__/*F*_IO_2__	324.9 ± 97.4	334.4 ± 102.5	304.0 ± 86.3	.422
Respiratory function				
RSBI, breath/min/L	122.8 ± 56.2	121.1 ± 61.1	126.7 ± 46.4	.799
Maximal inspiratory pressure, cm H_2_O	−36.5 ± 13.2	−35.2 ± 11.5	−39.2 ± 16.7	.438
Drive function				
Δ*P*_0.1_/Δ*P*_ETCO_2__, cm H_2_O/mmHg	0.33 ± 0.21	0.33 ± 0.22	0.34 ± 0.21	.991
$\;\Delta {\dot{V}}_{E}$/Δ*P*_ETCO_2__, L/min/mmHg	0.36 ± 0.16	0.40 ± 0.16	0.28 ± 0.12	.036^*∗*^

RSBI: rapid shallow breathing index; *∗* denotes statistical significance with *P* < .05.

**Table 3:** Multivariate analysis of possible factors with weaning success.

Factor	OR	95% CI	*P* value
Age	0.907	0.808–1.018	.097
Ventilation-use days till drive test	0.795	0.637–0.993	.043^*∗*^
Maximal inspiratory pressure	1.042	0.968–1.122	.273
Δ*P*_0.1_/Δ*P*_ETCO_2__	0.114	0.000–106.656	.534
$\Delta {\dot{V}}_{E}/\Delta P_{{ETCO}_{2}}$	6676.078	0.008–5.48*E* + 009	.205

Ventilation-use days till drive test: the duration time from the first day of mechanical ventilation use to the day of hypercapnic challenge test; *∗* denotes statistical significance with *P* < .05.

## Acronyms

PMV: Prolonged mechanical ventilation
RCC: Respiratory care center
ROC: Receiver operating characteristic
APACHE II: Acute Physiology and Chronic Health Evaluation II
AECOPD: Chronic obstructive pulmonary disease with acute exacerbation
RSBI: Rapid shallow breathing index
AUC: Area under the curve.

## References

[1] MacIntyre N. R., Epstein S. K., Carson S., Scheinhorn D., Christopher K., Muldoon S. (2005). Management of patients requiring prolonged mechanical ventilation: report of a NAMDRC consensus conference. *CHEST*.

[2] Kalil A. C., Metersky M. L., Klompas M. (2016). Management of Adults With Hospital-acquired and Ventilator-associated Pneumonia: 2016 Clinical Practice Guidelines by the Infectious Diseases Society of America and the American Thoracic Society. *Clinical Infectious Diseases*.

[3] Su J., Lin C.-Y., Chen P.-J., Lin F. J., Chen S.-K., Kuo H.-T. (2006). Experience with a step-down respiratory care center at a tertiary referral medical center in Taiwan. *Journal of Critical Care*.

[4] Boles J.-M., Bion J., Connors A. (2007). Weaning from mechanical ventilation. *European Respiratory Journal*.

[5] Tobin M. J. (1994). *Principles and Practice of Mechanical Ventilation*.

[6] Montgomery A. B., Holle R. H. O., Neagley S. R., Pierson D. J., Schoene R. B. (1987). Prediction of successful ventilator weaning using airway occlusion pressure and hypercapnic challenge. *CHEST*.

[7] Sassoon C. S. H., Mahutte C. K. (1993). Airway occlusion pressure and breathing pattern as predictors of weaning outcome. *American Review of Respiratory Disease*.

[8] Capdevila X. J., Perrigault P. F., Perey P. J., Roustan J. P. A., D'Athis F. (1995). Occlusion pressure and its ratio to maximum inspiratory pressure are useful predictors for successful extubation following T-piece weaning trial. *CHEST*.

[9] Wu Y.-K., Lee C.-H., Shia B.-C., Tsai Y.-H., Tsao T. C. Y. (2009). Response to hypercapnic challenge is associated with successful weaning from prolonged mechanical ventilation due to brain stem lesions. *Intensive Care Medicine*.

[10] Read D. J. (1967). A clinical method for assessing the ventilatory response to carbon dioxide. *Australasian Annals of Medicine*.

[11] Read D. J., Leigh J. (1967). Blood-brain tissue *P*_CO2_ relationships and ventilation during rebreathing. *Journal of Applied Physiology*.

[12] Mitchell R. A., Loeschcke H. H., Massion W. H., Severinghaus J. W. (1963). Respiratory responses mediated through superficial chemosensitive areas on the medulla. *Journal of Applied Physiology*.

[13] Ceorgopoulos D., Mitrouska I., Webster K., Bshouty Z., Younes M. (1997). Effects of inspiratory muscle unloading on the response of respiratory motor output to CO_2_. *American Journal of Respiratory and Critical Care Medicine*.

[14] Raurich J. M., Rialp G., Ibáñez J., Campillo C., Ayestarán I., Blanco C. (2008). Hypercapnia test as a predictor of success in spontaneous breathing trials and extubation. *Respiratory Care*.

[15] Raurich J. M., Rialp G., Ibáñez J., Llompart-Pou J. A., Ayestarán I. (2011). CO_2_ response and duration of weaning from mechanical ventilation. *Respiratory Care*.

[16] Solin P., Roebuck T., Johns D. P., Walters E. H., Naughton M. T. (2000). Peripheral and central ventilatory responses in central sleep apnea with and without congestive heart failure. *American Journal of Respiratory and Critical Care Medicine*.

[17] Jokic R., Zintel T., Sridhar G., Gallagher C. G., Fitzpatrick M. F. (2000). Ventilatory responses to hypercapnia and hypoxia in relatives of patients with the obesity hypoventilation syndrome. *Thorax*.

[18] Raurich J. M., Rialp G., Ibáñez J., Ayestarán I., Llompart-Pou J. A., Togores B. (2009). Hypercapnia test and weaning outcome from mechanical ventilation in COPD patients. *Anaesthesia and Intensive Care*.

[19] Murray J. F., Nadel J. A. (1988). *Textbook of Respiratory Medicine*.

[20] Pourriat J. L., Baud M., Lamberto C., Fosse J. P., Cupa M. (1992). Effects of doxapram on hypercapnic response during weaning from mechanical ventilation in COPD patients. *CHEST*.

[21] Peterson D. D., Pack A. I., Silage D. A., Fishman A. P. (1981). Effects of aging on ventilatory and occlusion pressure responses to hypoxia and hypercapnia. *The American Review of Respiratory Disease*.

[22] Sassoon C. S. H., Caiozzo V. J., Manka A., Sieck G. C. (2002). Altered diaphragm contractile properties with controlled mechanical ventilation. *Journal of Applied Physiology*.

[23] Jubran A., Tobin M. J. (1997). Pathophysiologic basis of acute respiratory distress in patients who fail a trial of weaning from mechanical ventilation. *American Journal of Respiratory and Critical Care Medicine*.

[24] Smith J., Bellemare F. (1987). Effect of lung volume on in vivo contraction characteristics of human diaphragm. *Journal of Applied Physiology*.

[25] Jones N. L., Robertson D. G., Kane J. W. (1979). Difference between end-tidal and arterial P_CO2_ in exercise. *Journal of Applied Physiology: Respiratory, Environmental And Exercise Physiology*.

[26] St Croix C. M., Cunningham D. A., Kowalchuk. J. M., et al. (1995). Estimation of arterial P_CO2_ in the elderly. *Journal of Applied Physiology*.

[27] Benallal H., Busso T. (2000). Analysis of end-tidal and arterial P_CO2_ gradients using a breathing model. *European Journal of Applied Physiology*.

[28] Hermans G., Agten A., Testelmans D., Decramer M., Gayan-Ramirez G. (2010). Increased duration of mechanical ventilation is associated with decreased diaphragmatic force: a prospective observational study. *Critical Care*.

[29] Powers S. K., Kavazis A. N., Levine S. (2009). Prolonged mechanical ventilation alters diaphragmatic structure and function. *Critical Care Medicine*.

[30] Dermot Frengley J., Sansone G. R., Shakya K., Kaner R. J. (2014). Prolonged mechanical ventilation in 540 seriously Ill older adults: effects of increasing age on clinical outcomes and survival. *Journal of the American Geriatrics Society*.

[31] Li J., Zhan Q. Y., Wang C. (2016). Survey of prolonged mechanical ventilation in intensive care units in mainland China. *Respiratory Care*.

